# The m6A methylation regulates gonadal sex differentiation in chicken embryo

**DOI:** 10.1186/s40104-022-00710-6

**Published:** 2022-05-18

**Authors:** Jianbo Li, Xiuan Zhang, Xiqiong Wang, Congjiao Sun, Jiangxia Zheng, Junying Li, Guoqiang Yi, Ning Yang

**Affiliations:** 1 National Engineering Laboratory for Animal Breeding and Key Laboratory of Animal Genetics, Breeding and Reproduction, Ministry of Agriculture and Rural Affairs, China Agricultural University, Beijing, China; 2 Shenzhen Branch, Guangdong Laboratory of Lingnan Modern Agriculture, Genome Analysis Laboratory of the Ministry of Agriculture and Rural Affairs, Agricultural Genomics Institute at Shenzhen, Chinese Academy of Agricultural Sciences, Shenzhen, China

**Keywords:** Chicken, Embryo, Gonads, m6A, YTHDC2

## Abstract

**Background:**

As a ubiquitous reversible epigenetic RNA modification, N6-methyladenosine (m6A) plays crucial regulatory roles in multiple biological pathways. However, its functional mechanisms in sex determination and differentiation during gonadal development of chicken embryos are not clear. Therefore, we established a transcriptome-wide m6A map in the female and male chicken left gonads of embryonic day 7 (E7) by methylated RNA immunoprecipitation sequencing (MeRIP-seq) to offer insight into the landscape of m6A methylation and investigate the post-transcriptional modification underlying gonadal differentiation.

**Results:**

The chicken embryonic gonadal transcriptome was extensively methylated. We found 15,191 and 16,111 m6A peaks in the female and male left gonads, respectively, which were mainly enriched in the coding sequence (CDS) and stop codon. Among these m6A peaks, we identified that 1013 and 751 were hypermethylated in females and males, respectively. These differential peaks covered 281 and 327 genes, such as *BMP2*, *SMAD2*, *SOX9* and *CYP19A1*, which were primarily associated with development, morphogenesis and sex differentiation by functional enrichment. Further analysis revealed that the m6A methylation level was positively correlated with gene expression abundance. Furthermore, we found that YTHDC2 could regulate the expression of sex-related genes, especially *HEMGN* and *SOX9*, in male mesonephros/gonad mingle cells, which was verified by in vitro experiments, suggesting a regulatory role of m6A methylation in chicken gonad differentiation.

**Conclusions:**

This work provided a comprehensive m6A methylation profile of chicken embryonic gonads and revealed YTHDC2 as a key regulator responsible for sex differentiation. Our results contribute to a better understanding of epigenetic factors involved in chicken sex determination and differentiation and to promoting the future development of sex manipulation in poultry industry.

**Supplementary Information:**

The online version contains supplementary material available at 10.1186/s40104-022-00710-6.

## Background

Sex manipulation technologies are of great significance to the production performance and economic benefit of livestock [1]. During the production of laying hens, at least seven billion day-old male chicks are culled every year worldwide, which raises cost and animal welfare concerns [2–4]. Research on the mechanisms of sex determination and differentiation in chickens, especially during the embryonic stage, will contribute to achieving early sex manipulation of chickens and be beneficial for chicken breeding and resource protection strategies [5]. In addition, chicken embryonic gonadal differentiation is also an excellent model for studying key factors of vertebrate sex determination [6] and human sexual development disorders [7, 8]. Although many investigations have focused on chicken sex determination and differentiation [9–20], the mechanisms underlying gonadal differentiation are still elusive.

The sex determination and differentiation of chickens during the embryonic stage is how the gonad develops into the testis or ovary. The process is well-known to be affected by the regulation of genetic factors and hormone levels [10, 12, 18, 20]. Unlike mammals, homogametic males (ZZ) produce testes, and heterogametic females (ZW) develop ovaries in birds. Considering that no female sex-determining genes have been found on the W chromosome [21], multiple studies have recognized that the Z chromosome gene doublesex and mab-3–related transcription factor 1 (*DMRT1*) is the key factor for the formation of testes [12, 16, 20]. The key conserved Sertoli cell differentiation factor (SOX9) may be induced and activated by high levels of DMRT1 in males (ZZ) and initiate testicular differentiation during embryonic development [16, 20, 22]. Due to the low dosage of DMRT1, the FOXL2 signaling pathway in females is activated [16, 20]. Estrogen is a key factor in determining female sex, and the blockade of estrogen could directly cause female embryonic gonad masculinization [9–11, 18, 20]. Although several genes responsible for sex determination and differentiation were identified, multiple pieces of evidence indicate that epigenetic modification is involved in this process [23–30]. The noncoding RNA transcribed from the male hypermethylated region (MHM) has been suggested to be associated with chicken gonadal development and differentiation [24, 25, 28–30]. Temporarily feminized gonads maintain transcriptomic and epigenetic memories of genetic sex [23]. Therefore, in-depth exploration from epigenetic aspects would provide novel insights into sex differentiation.

N6-methyladenosine (m6A) is an important posttranscriptional mRNA modification discovered in the 1970s and mainly regulates mRNA metabolism, including RNA stability, translation, RNA splicing, and transport [31–38]. In mammals, multiple studies have shown that m6A modification plays essential roles during spermatogenesis [39–43]. Knockout of *Mettl3*, *Mettl14* and *Ythdc2* in mice inhibited gonadal development and resulted in infertility [39–41]. Evidence has illustrated that m6A methylation is also involved in oocyte meiosis in *Xenopus laevis* [44]. *Ythdf2* and *Ythdf3* deletion prevented female gonad formation in zebrafish [45]. In addition, the dynamic m6A modification is abundant during follicle development in pigs [46] or follicle selection in chickens [47]. Several studies have shown that m6A methylation involves numerous biological processes [39–54]. However, knowledge about the distribution and function of m6A in chicken sex determination and differentiation of gonads is limited.

To decipher the role of RNA modification in the gonadal differentiation of chicken embryos, we performed global MeRIP-seq in female and male left gonads on E7. The present work generated a high-resolution m6A methylation profile and explored the potential molecular mechanisms underlying sex differentiation. We found that many differential m6A methylation peaks existed between the female and male left gonads in chicken embryos, and most genes with differential m6A methylation were involved mainly sex differentiation and development. It should be noted that *YTHDC2* can influence the expression of sex differentiation-related genes in males, which was confirmed by in vitro experiments. Our results provide distinctive insights into the epigenetic mechanism of chicken embryonic gonadal differentiation and will benefit further investigation of sex manipulation in poultry industry and research on human disorders of sexual development.

## Methods and materials

### Ethics statement

The experiments were approved by the Animal Welfare Committee of China Agricultural University and performed in accordance with the protocol outlined in the “Guide for Care and Use of Laboratory Animals” (China Agricultural University).

### Embryo tissue collection and RNA isolation

Fertilized eggs were obtained from a pure line of *White Leghorns* raised in the Experimental Base of Poultry Genetic Resources and Breeding, College of Animal Science and Technology, China Agricultural University. The eggs were incubated in an automated egg incubator at 37.8 °C and 60% relative humidity with rotation every 2 h. Once the eggs reached E7, only the left gonads of the chicken embryo were collected, immediately put into the RNAlater™ Stabilization Solution (Invitrogen, Carlsbad, CA, USA), and stored at − 20 °C. The remaining embryo tissues were used to determine sex by direct PCR kit (TransGen Biotech, Beijing, China) with *CHD1* primers (forward-5′-GTTACTGATTCGTCTACGAGA-3′, reverse-5′-TTGAAATGATCCAGTGCTTG-3′) [55]. Due to the RNA sample from a single individual gonad is not enough for MeRIP-seq, more than 1200 embryos were collected, and around 200 left gonads were mixed into a pool according to sex. Subsequently, six pools from three pools of female left gonads and three pools of males were prepared for RNA isolation. Total RNA was extracted with TRIzol reagent (Invitrogen, Carlsbad, CA, USA) following the manufacturer’s instructions. The RNA concentration and purity were measured using a NanoDrop 2000 spectrophotometer (Thermo Fisher Scientific, Waltham, MA, USA). The integrity of the RNA was determined using an Agilent 2100 Bioanalyzer (Agilent Technologies, CA, USA).

### MeRIP-seq

Poly(A) RNA was purified from 40 μg of total RNA using Dynabeads Oligo (dT) and then fragmented into 100-nucleotide-long fragments using Magnesium RNA Fragmentation Module (Illumina, Inc., CA, United States) at 86 °C for 7 min. Then, the fragmented mRNAs were incubated for 2 h at 4 °C with anti-m6A polyclonal antibody (Synaptic Systems, Goettingen, Germany) in IP buffer. Eluted m6A-containing fragments (IP) and untreated input control fragments were then concentrated to generate the final cDNA library. The libraries were qualified and absolutely quantified using an Agilent Bioanalyzer 2100 (Agilent Technologies, CA, USA). The prepared libraries were then sequenced on an Illumina NovaSeq 6000 (150 bp paired-end, PE150).

### The analysis of sequencing data

The clean reads were aligned to the chicken genome sequences (GRCg6a) with HISAT2 [56]. SAM files were converted to the BAM format using Samtools [57], and PCR duplicates were removed using the Picard MarkDuplicates option to generate filtered BAM files. The m6A modification peaks were called using MACS2 [58] (−g 9.6e8 --nomodel --extsize 200 -q 0.05). Meanwhile, input data (high-throughput paired-end RNA sequencing, RNA-seq) were used as background. Referring to the proportion of the effective genome sizes of humans and mice, we adjusted the parameters of -g in chickens. The number of reads falling in the m6A peak in each sample was counted with bedtools [59]. Moreover, the putative peaks were annotated, and the motifs enriched in peak regions were analyzed using RNAmod [60]. The Htseq-count [61] tool was used to count the gene-level reads. DEGs were identified with the R package DESeq2 [62] between female and male gonads. The genes/peaks with fold change > 2 or < 0.5 and FDR < 0.05 were considered different.

### Functional annotation

Using BioMart [63], we identified homologs of chicken DEGs in humans. Functional analysis of these homologs was performed using the Metascape online tool [64]. The Gene Ontology (GO) terms for biological process, cellular component, and molecular function categories, as well as Kyoto Encyclopedia of Genes and Genomes (KEGG) pathways, were enriched based on the Metascape online tool with default parameters.

### Cell separation and culture

The tissues (mesonephros and gonads) were harvested and placed in PBS when the fertile chicken eggs reached E7. Samples were dispersed by incubation with trypsin 0.25% (GIBCO, Grand Island, NY, USA) at 37 °C for 10 min with constant shaking. The digestions were stopped by the addition of culture medium containing DMEM/F12 with 10% (v/v) fetal bovine serum and 1% penicillin/streptomycin (GIBCO, Grand Island, NY, USA), and the samples were passed through a cell strainer (40 μm) and collected into 50-mL tubes. Subsequently, the cells were washed with PBS and seeded in cell culture plates. Sex identification was carried out by PCR as described above. All the cells from individual males were mixed.

### Small interfering RNA assays and qRT-PCR

The design and synthesis of *YTHDC2*-siRNA primer was performed by RiboBio (Guangzhou, China). For the *YTHDC2*-knockdown assay, the following sequence was used: 5′-CTCACAGATACCAAGTAT-3′. We applied *YTHDC2*-siRNA (RiboBio, Guangzhou, China) according to Fugene HD (Promega, Madison, WI, USA) transfection into male cells. After transfection for 48 h, the cells were collected for RNA extraction, and cDNA was synthesized using the First-Strand Synthesis kit (Takara, Japan) and PrimeScript™ RT reagent kit with gDNA Eraser (Takara, Japan). Gonadal cDNA was also synthesized as described above. Quantitative real-time polymerase chain reaction (qRT-PCR) was performed with an ABI 7500 system (Applied Biosystems, Bedford, MA, USA) using the TB Green® Premix Ex Taq™ Kit (Takara, Japan) according to the manufacturer’s instructions. Primer sequences for *β-actin*, *AMH*, *SOX9*, *FOXL2* and *CYP19A1* have been published by our previous results [65]. The primer of *YTHDC2*, *FTO*, *METTL3*, *ALKBH5*, *DMRT1* and *HEMGN* were designed by DNAMAN version 6.0 software. *β-actin* was used as the internal control, and the sequences of the gene-specific primers are listed in Table 1.

**Table 1:** Primers used in qRT-PCR

Gene	Primer	Accession number	Annealing temperature, °C	Fragment size, bp
*β-actin*	F:5′- GAGAAATTGTGCGTGACATCA- 3'	NM_205518	54/56	152
	R:5′- CCTGAACCTCTCATTGCCA- 3'			
*YTHDC2*	F: 5′- GATGTCGTTTCCTTCGTC- 3'	XM_004949271	54	187
	R: 5′- CTGTTTCGTTCTGGGTGT- 3'			
*FTO*	F: 5′- GGGACATAGAGACACCTG- 3'	NM_001185147	54	253
	R: 5′- GCAGTTTCCAGTGATTTC- 3'			
*METTL3*	F: 5′- TAAGTTCGCCGTGGTGAT- 3'	XM_025145967	54	181
	R: 5′- CCAAAGGTTCAGGCATTC- 3'			
*ALKBH5*	F: 5′- CGCTGCGGAACAAGTATT- 3'	NM_001257201	54	282
	R: 5′- TGAAGAACGACACGGAGA- 3'			
*DMRT1*	F: 5′- GCAACCACGGCTACTCCTCGC- 3'	NM_001101831	56	146
	R: 5′- TTCCTGGGCTTGCTGCCTCCT- 3'			
*HEMGN*	F: 5′- AACCACAGCCAAACCCTC- 3'	XM_430508	56	185
	R: 5′- CAGCATATCCTCTTCACCC- 3'			
*AMH*	F: 5′- GGATGGAGGTGCCCCTCTGT- 3'	NM_205030	56	129
	R: 5′- GCAGCATCACCCTCAGGTGG- 3'			
*SOX9*	F: 5′- AGTACCCGCATCTGCACAA- 3'	NM_204281	56	161
	R: 5′- CCTCCTGCGTGGTTGGTA- 3'			
*FOXL2*	F: 5′- CTGATCGCCATGGCCATACG- 3'	NM_001012612	56	127
	R: 5′- GGCGGATGCTGTTCTGCCA- 3'			
*CYP19A1*	F: 5′- GGAATTGGGCCTCTCATTTC- 3'	NM_001364699	56	154
	R: 5′- CGTGAAATACGCTGGAGGAT- 3'			

Abbreviations: *F* Forward, *R* Reverse

### Statistical analysis

In this study, statistical analyses were performed with SPSS 22.0 (SPSS, Chicago, IL, USA). The data are expressed as the mean ± SD (standard deviation) and were analyzed using a two-tailed Student’s *t*-test, and at least three replicates were conducted in multiple independent experiments. The differences were considered to be statistically significant at a *P*-value < 0.05.

## Results

### General features of m6A methylation in chicken embryonic gonads

**Fig. 1:**
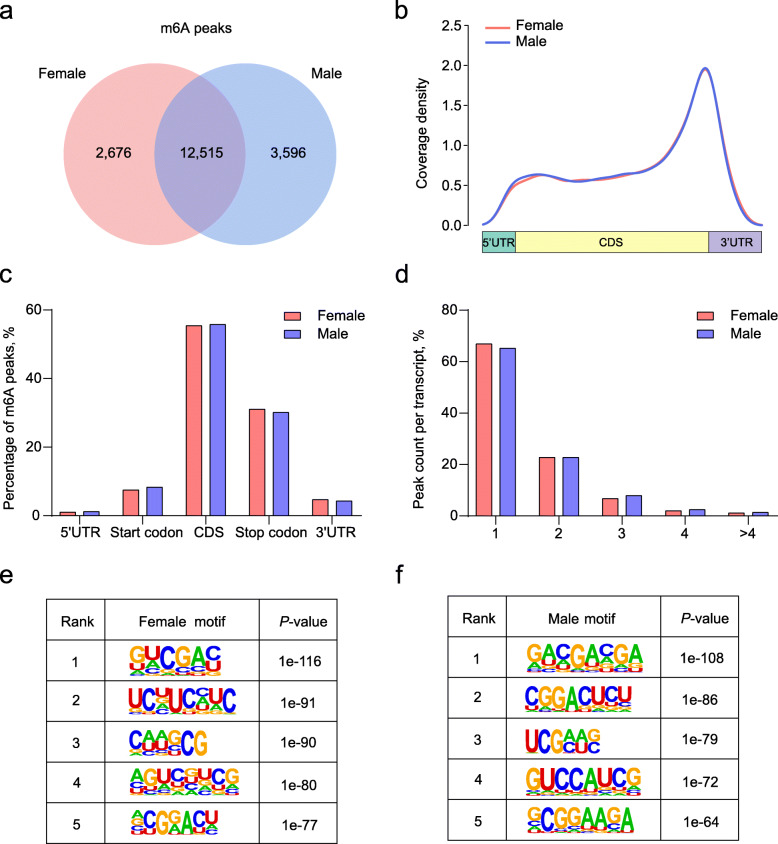
Overview of the m6A methylation profile in chicken embryonic gonads. **a** The overlap of m6A peaks between female and male gonads. **b** Distribution of m6A peaks along transcripts. Transcripts are grouped into 5′ UTR, CDS, and 3′ UTR methylation based on the identified m6A sites. **c** Analysis of gonadal m6A peaks in different genomic features. **d** Percentage of m6A methylated transcripts with different numbers of m6A peaks. **e, f** The top motifs enriched across m6A peaks identified from female and male gonads

Our previous RNA-seq data indicated that *YTHDC2* showed differential expression levels between females and males in early chicken embryonic gonads (Additional file 1: Fig. S1a). Therefore, we hypothesized that m6A methylation may play significant roles in the process of sex determination and differentiation. To gain insight into this phenomenon, we collected the left gonads of both sexes at E7 in triplicate for MeRIP-seq. The mapping statistics of MeRIP-seq from all samples are displayed in Additional file 2: Table S1, suggesting that each sample showed high quality for the following analyses. We identified 15,191 and 16,111 m6A peaks in the female and male left gonads in chicken embryos, respectively. Overall, 12,515 m6A peaks overlapped between the two groups, along with 2676 and 3596 unique m6A peaks in female and male left gonads (Fig. 1a), indicating that m6A methylation in the male gonadal gene is relatively richer. Consistent with previous studies [66], gonadal m6A methylation peaks were generally found in the genomic features, in which exon regions accounted for the largest proportion (Additional file 1: Fig. S1b).

To further refine the topological pattern of m6A modification in chicken embryonic gonads, we investigated the distribution of m6A peaks. The results demonstrated that the peaks were markedly enriched in the CDS and stop codon (Fig. 1b, c). Based on the number of m6A peaks contained in each transcript, we further classified the methylated transcripts into five types and found that approximately 90% of transcripts contained one or two m6A peaks (Fig. 1d). Overall, there was high consistency in the distribution of m6A peaks between female and male chicken embryonic left gonads. Given that the motifs that bind to various transcription factors are the sites where RNA methylation and demethylation begin, we performed a comprehensive motif scan analysis on male and female m6A peaks, respectively. We found that GGACU was significantly enriched in the female and male chicken embryonic left gonads (Fig. 1e, f), which is in agreement with previous studies [67, 68].

### Differential m6A methylation analysis

**Fig. 2:**
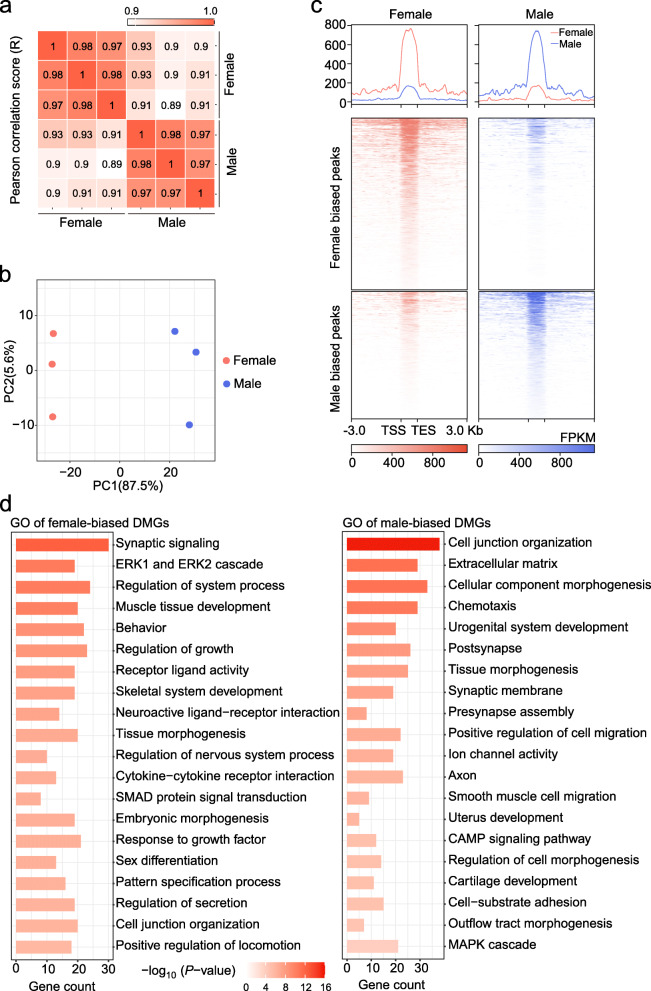
Analysis of differential m6A methylation in chicken embryonic gonads. **a** A heatmap of the sample correlation matrix of m6A showing high similarities between duplicates and dissimilarities between female and male left gonads. **b** PCA plot of MeRIP-seq of female and male left gonads. **c** Bandplots (top) and heatmaps (bottom) showing the quantification of MeRIP-seq data of female-biased and male-biased m6A peaks in gonads at E7. **d** Top 20 significantly enriched terms of female-biased (left) and male-biased (right) DMGs

Three replicates of MeRIP-seq in the female and male groups showed high concordance using the Pearson correlation coefficient (Fig. 2a). A principal component analysis (PCA) plot based on the m6A methylation level displayed a clear separation between female and male left gonads (Fig. 2b), in which PC1 explained 87.5% of the phenotypic variance. To detect the differences in m6A methylation levels between female and male gonads, we assessed the differentially methylated m6A peaks (DMPs) marked by MeRIP signals. A total of 1764 DMPs were detected, including 1013 female- and 751 male-biased DMPs in chicken embryonic left gonads (Fig. 2c), which corresponded to 281 and 327 protein-coding genes (DMGs; Additional file 2: Table S2). The heatmap revealed that these sex-biased DMPs showed highly clear sex-specific patterns at E7 (Fig. 2c). The top 20 female- and male-biased DMPs are listed in Additional file 2: Table S3. The annotation results of DMPs on the chromosome distribution indicated that 56.3%, 1.7%, 39.0% and 3.1% female-biased peaks and 72.7%, 1.1%, 0.5% and 25.7% male-biased peaks were located on the autosome, unknown chromosome, W chromosome and Z chromosome (Additional file 1: Fig. S1c). More DMPs were located on Z chromosome in males than in females.

The distribution of DMGs on chromosomes was similar between female and male gonads (Additional file 1: Fig. S1d). Among these DMGs, we found that some genes were related to sex determination and differentiation, such as *FOXL2*, *CYP19A1*, *AMH* and *SOX9*. In addition, many development-related genes were also discovered, such as *BMP2*, *BMPR2*, *FGF2* and *SMAD2*. To better understand the functional consequences of m6A methylation, we performed a functional enrichment analysis of sex-biased DMGs using Metascape software. The top 20 significantly enriched gene ontology (GO) terms were identified (Fig. 2d). The significant terms of the female-biased DMPs were related to ERK1 and ERK2 cascade, muscle tissue development, regulation of growth, receptor ligand activity, response to growth factor, sex differentiation, tissue morphogenesis, SMAD protein signal transduction and embryonic morphogenesis. Moreover, the terms driven by the male-biased DMPs were enriched mainly in cell junction organization, cellular component morphogenesis, urogenital system development, tissue morphogenesis, positive regulation of cell migration, MAPK cascade, smooth muscle cell migration, outflow tract morphogenesis, regulation of cell morphogenesis.

### Correlation analysis of m6A methylation and DEGs

**Fig. 3:**
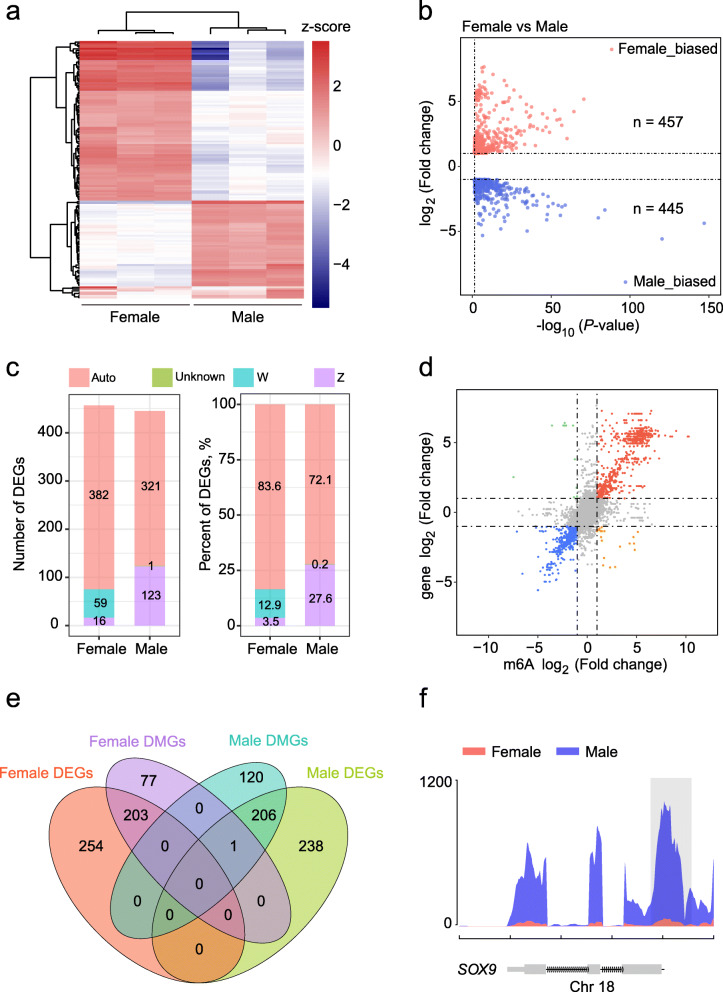
Integration analysis of MeRIP-seq and RNA-seq. **a** Unsupervised clustering analysis showing the expression profiles of the top 1000 most variable genes in the female and male gonads at E7. **b** Volcano plots of DEGs number between female and male left gonads. **c** The number and percentage of DEGs in each chromosomal allocation between female and male gonads. **d** Four-quadrant graph exhibiting the genes containing methylated m6A peaks in chicken embryonic gonads. **e** Venn diagrams show the shared and unique genes obtained from four groups: female DMGs, male DMGs, female DEGs and male DEGs. **f** The abundance of m6A peaks in the *SOX9* gene of female and male gonads detected by MeRIP-seq

**Fig. 4:**
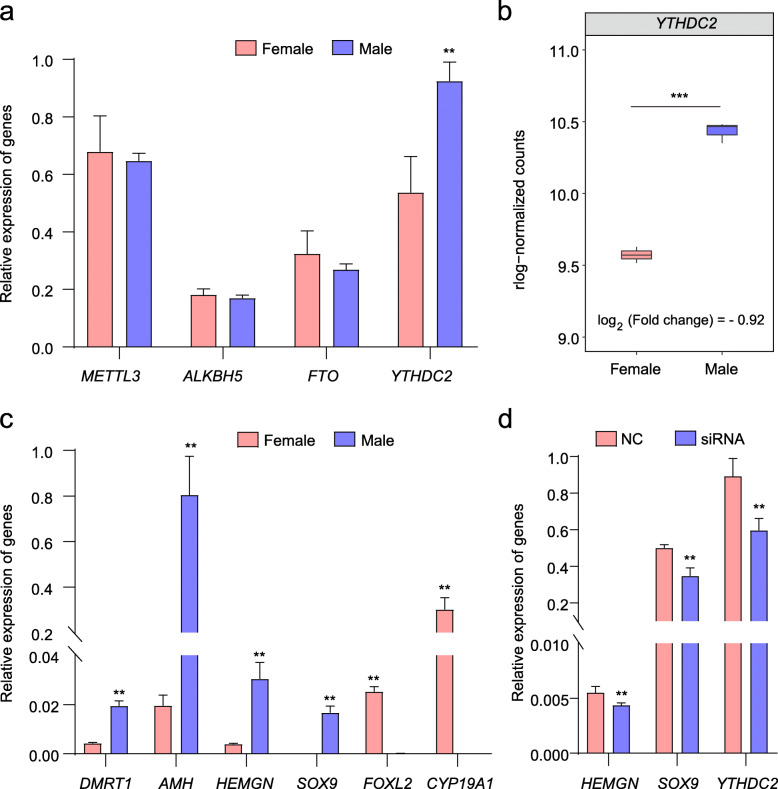
Comprehensive analysis of gene expression. **a** qRT-PCR results of m6A methylation-related genes in chicken embryonic gonads. **b** The expression of *YTHDC2* at E7 in chicken embryonic gonads. The y axis shows the rlog-normalized counts. **c** qRT-PCR results of sex differentiation-related genes in chicken embryonic gonads. **d** Changes in the relative expression of genes related to male gonadal markers in cells with *YTHDC2* knockdown. * and ** indicate *P* < 0.05 and *P* < 0.01, respectively

To clarify the functional consequences of the gene expression levels modified by m6A methylation, we profiled the global transcriptomic landscape of both gonads on the left using RNA-seq. The results of sequencing alignment of RNA-seq are listed in Additional file 2: Table S1. Similar to the m6A results, unsupervised hierarchical clustering of the top 1000 most variable genes revealed a distinct expression signature in female and male left gonads (Fig. 3a). RNA-seq analysis detected 902 differentially expressed genes (DEGs), including 457 female- and 445 male-biased DEGs (Fig. 3b). The majority of DEGs resided on the autosome (Fig. 3c). Subsequently, the global relationship between gene expression and m6A methylation was calculated. By assigning m6A methylation regions to the nearest genes, we found that most genes showed a positive correlation between the expression levels and the abundance of m6A peaks based on calculated fold changes, and only a few of them were negatively correlated (Fig. 3d). Moreover, we found 410 DEGs with differential m6A methylation levels. A total of 203 and 206 genes with m6A hypermethylation showed upregulated transcription levels in females and males, respectively, and one gene with m6A hypomethylation was downregulated in females (Fig. 3e). Interestingly, many sex-related genes showed differences in both m6A methylation and mRNA expression (Fig. 3f, Additional file 1: Fig. S1e-h, Fig. 4c). For instance, *SOX9*, a male gonadal developmental marker gene, showed significantly higher m6A methylation and mRNA levels in the male gonads (Figs. 3f, 4c), while *CYP19A1*, which is associated with female gonadal differentiation, exhibited stronger m6A methylation and mRNA expression levels in the female gonads (Additional file 1: Fig. S1h, Fig. 4c). Additionally, the genes of *HEMGN*, *AMH* and *FOXL2* all have DMPs between female and male gonads (Additional file 1: Fig. S1e-g); however, we did not find DMPs in the *DMRT1* gene body.

To explore the regulatory mechanism of m6A methylation in chicken gonadal differentiation, we performed a series of in vitro experiments for some differential m6A methylation-related genes and genes involved in gonadal differentiation. We found that the expression level of *YTHDC2* was significantly higher in male gonads (Fig. 4a), which is consistent with the RNA-seq results (Fig. 4b). Some marker genes of sex differentiation were also differentially expressed (Fig. 4c). Moreover, we found that the stop codon of *SOX9* and the CDS of *HEMGN* showed significant enrichment in IP samples (Fig. 3f; Additional file 1: Fig. S1e). Based on these results, we hypothesize that sex-related genes are affected by YTHDC2. To clarify the process, we used *YTHDC2*-siRNA to downregulate the expression of *YTHDC2* in male mesonephros/gonad mingle cells. Interestingly, the expression of the sex-related genes *SOX9* and *HEMGN* was downregulated in the *YTHDC2*-siRNA group (Fig. 4d). Based on these observations, we preliminarily concluded that YTHDC2 could affect sex differentiation-related gene expression by regulating epigenetic patterns in chicken embryonic gonads. Therefore, m6A methylation may play a role in sex determination and differentiation in chicken.

## Discussion

The chicken embryo is a superior model in the study of cell biology and organ development [6, 69–74]. In the past few decades, the regulatory mechanisms for chicken sex determination and differentiation, such as transcription factors [12, 16, 20], DNA methylation [24, 25, 28–30] and hormones [9, 10, 18, 20, 23, 27], have been extensively studied. Recently, m6A methylation, a reversible apparent modification [31], has been reported to play an essential role in vertebrate physiology and reproductive processes like development, spermatogenesis, oogenesis and infertility [39–42, 44–50]. Notably, m6A methylation is essential in the sex determination and gonadal development process of many animals, including mice [39–41], fish [45, 75] and Drosophila [51–53]. However, the roles and molecular mechanisms of m6A modification in the sex differentiation of chicken embryos remain unclear. Here, we established a comprehensive m6A methylation profile in chicken embryonic gonads. Our results suggested that m6A methylation is significantly different between female and male gonads, and results in distinct expression patterns of many sex-related genes. Further, these marker genes involved in gonadal differentiation were confirmed to be regulated by m6A enzyme YTHDC2, which means that m6A methylation may be an important factor affecting chicken sex differentiation.

We found more m6A peaks in the male gonads than in the female gonads, implying that m6A methylation might be a vital impetus in sex determination and differentiation. The genomic distribution of m6A peaks in male and female embryonic gonads is globally similar. The percentage of m6A peaks located in the CDS was the largest, while the coverage density of m6A peaks was the highest at the stop codon in chicken embryonic gonads, which was consistent with research on mammals [67, 68, 76, 77]. Subsequently, we compared the DMPs between female and male gonads in chicken embryos. The quantitative advantage of DMPs in females is reflected mainly in the sex chromosomes, while the number of DMGs is equal between males and females, which may be caused by the poor annotation of the W chromosome [14]. Previous studies have also proven that m6A methylation plays an important role in the process of tissue development [78–82]. In this study, GO analysis showed that DMGs were enriched mainly in sex differentiation- and development-relevant items, including sex differentiation, SMAD protein signal transduction, urogenital system development and tissue morphogenesis, which suggests that DMGs were related to the dynamic process of gonadal development. A number of studies have reported the functions of these DMGs, like *BMP2* [83], *CYP19A1* [27], *ESR1* [18], *FOXL2* [6], *JUN* [84], *SMAD2* [84, 85] and *SOX9* [16, 20, 22]. Our findings revealed that the post-transcriptional modifications of these genes should be alternative factor driving sex difference, which means that m6A methylation may play a major role in the differentiation of gonads. This finding would contribute to understanding the mechanisms of sex determination and differentiation in chickens and providing a promising reference for further research of human gonad development related diseases.

The role and underlying mechanism of m6A modification in regulatory of gene expression is still uncharted territory. Collectively, m6A has been linked to reduce mRNA stability and promote mRNA degradation in various biological pathways [32, 86], suggesting that the m6A methylation level is negatively correlated with gene expression. In addition, several researches suggested a possible positive relationship between the extent of m6A methylation and the mRNA levels [87–90]. Integration analysis of RNA-seq and MeRIP-seq data in our work found that the expression level of most genes was positively correlated with the m6A methylation signal in the whole genome, which is also supported by many previous studies [87–90]. These findings mean that the roles of m6A methylation in transcriptional regulation need to be elucidated in future studies. The mRNA m6A modifications are well-known to be recognized and bound by m6A reader proteins, such as YTHDF1–3, YTHDC1 and YTHDC2 [36, 39, 45, 86, 91, 92]. In our study, RNA-seq analysis suggested that only *YTHDC2* was differentially expressed between female and male gonads among m6A methylation-related genes, which was verified by qRT-PCR. Previous studies have shown that the loss of YTHDC2 downregulates the meiotic genes in mitotic spermatogonia [42]. The function of YTHDC2 can reduce the translation efficiency of target genes and reduce the mRNA abundance in the meiosis of germline cells [39]. Interestingly, our in vitro experiments of *YTHDC2* knockdown in males suggested that *YTHDC2* could downregulate *HEMGN* and *SOX9* gene expression by reshaping their m6A patterns. HEMGN has been shown to be involved in sex determination in early chicken embryos [15]. *SOX9* is a marker gene of testicular differentiation during chicken embryonic development [16, 20, 22]. Nearly half of the DEGs showed differential m6A methylation modifications in the gene body, which were involved in gonadal differentiation and development terms. The expression of sex differentiation marker genes was also verified by qRT-PCR. In chicken, the changes of these candidate regulators of gonadal sex differentiation induced sex reversal [9–12, 15–17]. Thus, m6A methylation may play roles during sex differentiation in chickens. However, further research at the single-cell level is required to validate this hypothesis and will be helpful to elucidate molecular basis underlying gonadal differentiation. Here, we provided a pioneering work demonstrating the function of YTHDC2 in chicken embryonic gonads. Although the roles of YTHDC2 have been studied in mammals [39, 42, 43, 54, 76, 93, 94], the precise transcriptional regulation molecular mechanism underlying multiple crucial roles of YTHDC2 remains to be determined in chicken. More evidence is needed to explore whether YTHDC2 can directly regulate the expression of sex-related genes.

## Conclusions

This study first analyzed transcriptome-wide m6A methylation modification pattern in the gonads of chicken embryos and preliminarily explored roles of YTHDC2 in regulating key genes underlying sex differentiation. The m6A methylation profile and molecular basis underlying gonadal differentiation in chicken embryo provide a new avenue for studying RNA modification in vertebrate sex determination and differentiation and offer distinctive insights into the epigenetics mechanism of studies about human gonadal development and infertility.

## Supplementary Information

**Additional file 1: Fig. S1.** Characteristics of MeRIP-seq in chicken gonads. **a** Transcriptional change in *YTHDC2* between the female and male gonads at four developmental stages: (embryonic Day 4.5, E4.5), E5.5, E7 and E10. **b** The distribution of sequencing reads on the genome. **c-d** Percentage of DMPs (c) and DMGs (d) in each chromosomal allocation between female and male gonads. **e-h** The abundance of m6A peaks in the *HEMGN*, *AMH*, *FOXL2* and *CYP19A1* genes of female and male gonads detected by MeRIP-seq.

**Additional file 2: Table S1.** Summary of sequence data and read alignment statistics. **Table S2.** Summary of m6A peaks and annotation genes. **Table S3.** Summary of the female-biased and male-biased DMPs in chicken embryonic gonads.

## Data Availability

The sequencing datasets in this study are available at NCBI [PRJNA766306].

## Abbreviations

CDS: Coding sequence
DEGs: Differentially expressed genes
DMGs: Differential methylation related coding genes
DMPs: Differential methylation m6A peaks
E7: Embryonic Day 7
GO: Gene ontology
m6A: N6-methyladenosine
MeRIP-seq: Methylated RNA immunoprecipitation sequencing
PCA: Principal component analysis
RNA-seq: High-throughput paired-end RNA sequencing
