# Supplementary figures and images for: The m6A methylation regulates gonadal sex differentiation in chicken embryo
Source: J Anim Sci Biotechnol. 2022 May 18;13:52. doi: 10.1186/s40104-022-00710-6 (PMC9115958; doi:10.1186/s40104-022-00710-6)

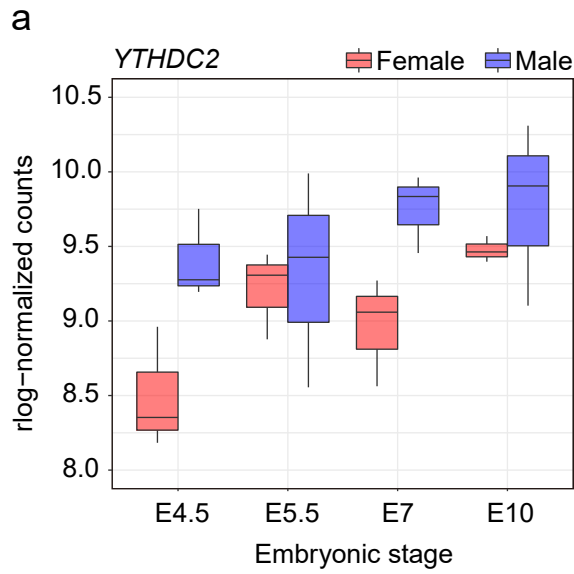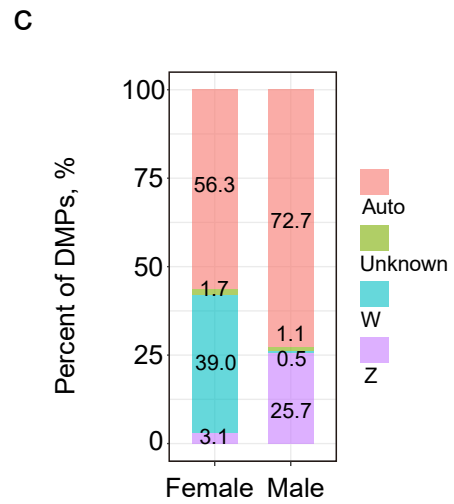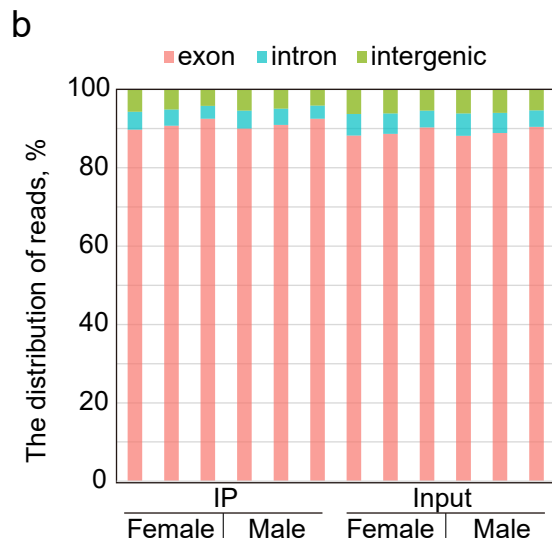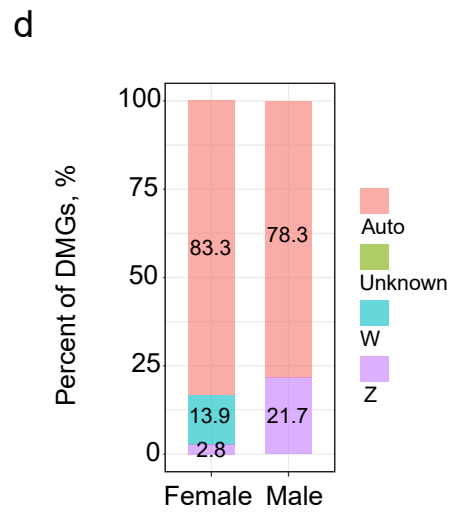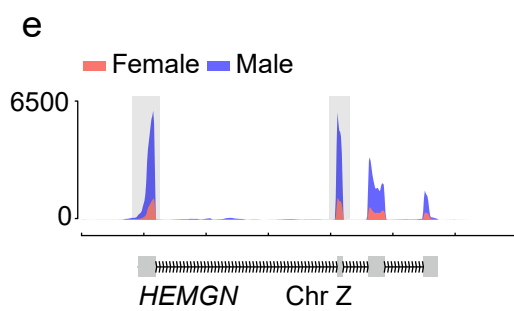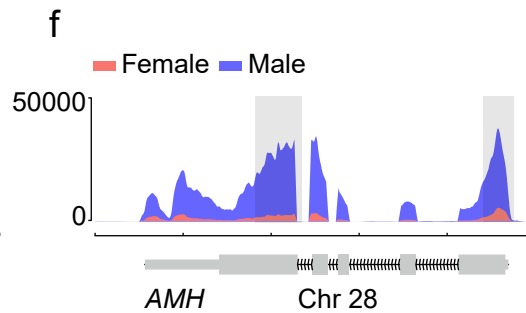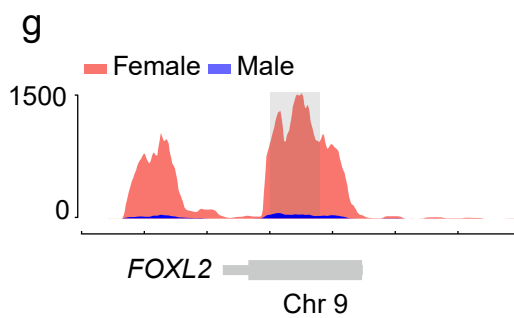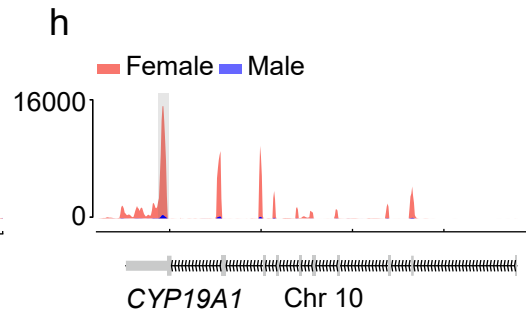

Supplement: Supplementary file 1 — Additional file 1: Fig. S1. Characteristics of MeRIP-seq in chicken gonads. a Transcriptional change in YTHDC2 between the female and male gonads at four developmental stages: (embryonic Day 4.5, E4.5), E5.5, E7 and E10. b The distribution of sequencing reads on the genome. c-d Percentage of DMPs (c) and DMGs (d) in each chromosomal allocation between female and male gonads. e-h The abundance of m6A peaks in the HEMGN, AMH, FOXL2 and CYP19A1 genes of female and male gonads detected by MeRIP-seq. [file 40104_2022_710_MOESM1_ESM.pdf]
